# Development and validation of a prognostic 40-day mortality risk model among hospitalized patients with COVID-19

**DOI:** 10.1371/journal.pone.0255228

**Published:** 2021-07-30

**Authors:** Donald A. Berry, Andrew Ip, Brett E. Lewis, Scott M. Berry, Nicholas S. Berry, Mary MrKulic, Virginia Gadalla, Burcu Sat, Kristen Wright, Michelle Serna, Rashmi Unawane, Katerina Trpeski, Michael Koropsak, Puneet Kaur, Zachary Sica, Andrew McConnell, Urszula Bednarz, Michael Marafelias, Andre H. Goy, Andrew L. Pecora, Ihor S. Sawczuk, Stuart L. Goldberg

**Affiliations:** 1 Berry Consultants LLC, Austin, Texas, United States of America; 2 M.D. Anderson Cancer Center of the University of Texas, Houston, Texas, United States of America; 3 Division of Outcomes and Value Research, John Theurer Cancer Center at Hackensack University Medical Center, Hackensack, New Jersey, United States of America; 4 John Theurer Cancer Center at Hackensack University Medical Center, Hackensack, New Jersey, United States of America; 5 Hackensack Meridian Health School of Medicine, Nutley, New Jersey, United States of America; 6 Hackensack Meridian Health, Edison, New Jersey, United States of America; Universita degli Studi di Napoli Federico II, ITALY

## Abstract

**Objectives:**

The development of a prognostic mortality risk model for hospitalized COVID-19 patients may facilitate patient treatment planning, comparisons of therapeutic strategies, and public health preparations.

**Methods:**

We retrospectively reviewed the electronic health records of patients hospitalized within a 13-hospital New Jersey USA network between March 1, 2020 and April 22, 2020 with positive polymerase chain reaction results for SARS-CoV-2, with follow-up through May 29, 2020. With death or hospital discharge by day 40 as the primary endpoint, we used univariate followed by stepwise multivariate proportional hazard models to develop a risk score on one-half the data set, validated on the remainder, and converted the risk score into a patient-level predictive probability of 40-day mortality based on the combined dataset.

**Results:**

The study population consisted of 3123 hospitalized COVID-19 patients; median age 63 years; 60% were men; 42% had >3 coexisting conditions. 713 (23%) patients died within 40 days of hospitalization for COVID-19. From 22 potential candidate factors 6 were found to be independent predictors of mortality and were included in the risk score model: age, respiratory rate ≥25/minute upon hospital presentation, oxygenation <94% on hospital presentation, and pre-hospital comorbidities of hypertension, coronary artery disease, or chronic renal disease. The risk score was highly prognostic of mortality in a training set and confirmatory set yielding in the combined dataset a hazard ratio of 1.80 (95% CI, 1.72, 1.87) for one unit increases. Using observed mortality within 20 equally sized bins of risk scores, a predictive model for an individual’s 40-day risk of mortality was generated as -14.258 + 13.460*RS + 1.585*(RS–2.524)^2–0.403*(RS–2.524)^3. An online calculator of this 40-day COVID-19 mortality risk score is available at www.HackensackMeridianHealth.org/CovidRS.

**Conclusions:**

A risk score using six variables is able to prognosticate mortality within 40-days of hospitalization for COVID-19.

**Trial registration:**

Clinicaltrials.gov Identifier: NCT04347993.

## Introduction

Although infection with the novel coronavirus SARS-CoV-2, the causative agent for COVID-19, may result in asymptomatic or minimally symptomatic illness, a significant proportion of individuals will require hospitalization and critical care support [1]. A striking observation drawn from epidemiologic studies has been that severe COVID-19 disease has occurred principally among individuals with pre-existing comorbid conditions [2]. A report from the US Centers for Disease Control and Prevention noted that 38% of COVID-19 patients had one or more underlying conditions. Patients with comorbidities were more likely to require hospitalization (71%) and intensive care support (78%) compared to individuals without additional risk factors (27%) [3]. High case fatality rates have been reported particularly among the elderly, nursing home populations, and individuals with pre-existing comorbid conditions [1–3]. As of June 3, 2021 in New York state, 91.7% (39,234 out of 42,745) of COVID-19 related deaths have occurred in patients with at least one comorbidity, with hypertension (54% of cases), diabetes (34%) and hyperlipidemia (22%), dementia (14%), coronary artery disease (13%), renal disease (11%) and chronic obstructive pulmonary disease (11%) being most common [4].

The ability to predict survival upon entry to the hospital, based on pre-existing comorbidities and presenting features, would permit healthcare teams to strategize individual treatment planning, more accurately evaluate the efficacy of new therapies, and assist in public health resource allocations. Well validated mortality models incorporating comorbidities and presenting features, similar to the APACHE II, SAPS II, and SOFA models used to prognosticate intensive care unit survival have been lacking for COVID-19 [5–7]. A systematic review identified 23 proposed prognostic survival models but concluded that these all were at high risk of bias, mainly because of non-representative selection of control patients, exclusion of patients who had not experienced the event of interest by the end of the study, and model overfitting [8].

One of the first validated survival models was reported by the National Health Commission of the People’s Republic of China and included 10 independent predictive factors (chest radiographic abnormality, age, hemoptysis, dyspnea, unconsciousness, number of comorbidities, cancer history, neutrophil-to-lymphocyte ratio, lactate dehydrogenase, and direct bilirubin) [9]. However, this prognostic model included only 59 deaths and has not been externally validated in western societies with differing patient characteristics and healthcare delivery capabilities. Among the expanding list of other models one of the largest was reported by investigators in the United Kingdom who gathered observational data from 260 hospitals across England, Scotland, and Wales on 57,824 COVID patients with a mortality rate of 31.4%. Their 4C Mortality Score included eight variables (age, sex, number of comorbidities, respiratory rate, peripheral oxygen saturation, level of consciousness, urea level, and C reactive protein) [10]. Another approach, from Spanish investigators, focused on prognostic features directly associated COVID-19 pathogenesis, rather than patient characteristics, to build a mortality model based on peripheral oxygenation level, neutrophil count, platelet count, lactate dehydrogenase, and C-reactive protein levels at the time of hospitalization [11]. Although these models all share some common variables, the differences are notable.

We sought to develop and validate a prognostic mortality model that incorporated both pre-existing comorbidities and presenting features among a USA population of hospitalized COVID-19 patients. Through April 22, 2020 Hackensack Meridian Healthcare’s network of 13 hospitals within New Jersey had provided care to over 3000 COVID-19 patients and had experienced over 700 deaths. Using this cohort we present a new model that prognosticates the risk of mortality within 40 days of hospitalization for COVID-19 illness.

## Materials and methods

### Study design and cohort selection

This is a retrospective, observational, multicenter cohort study. Our primary objective was to develop and internally validate a prognostic mortality risk model.

Hackensack Meridian Health network (HMH) had established an observational database drawn from the electronic health records (EHR) of hospitalized COVID-19 patients as described below (Clinicaltrials.gov Identifier: NCT04347993). Patients were included in the database based on the following inclusion and exclusion criteria: 1) Positive SARS-CoV-2 diagnosis by reverse-transcriptase polymerase chain reaction and 2) Hospitalization at one of HMH’s 13-hospitals within the time frame of March 1, 2020 until April 22, 2020. For the purposes of generating the mortality-risk model we also excluded 1) pregnancy, 2) enrollment in a randomized clinical trial, and 3) died on the day of admission to the hospital. We accessed the data on June 10, 2020.

Institutional Review Board (IRB) approval was obtained for access to the prospective observational database. Informed consent was waived by the IRB as this project represented a non-interventional study utilizing de-identified data within the database.

### Data sources

We collected data from HMH’s EHR (Epic, Verona WI) which is utilized throughout the network. Hospitalized patients throughout HMH were flagged by the EHR if SARS-CoV-2 testing was positive. These EHR-generated reports served as our eligible cohort to sample. Demographic, clinical characteristics, treatments, and outcomes were manually abstracted by research nurses and physicians from the John Theurer Cancer Center at Hackensack University Medical Center. Assigning patients to our data team occurred in real-time, and not randomized. Data abstracted by the team were entered using REDCap (Research Electronic Data Capture, Vanderbilt University). Quality control was performed by physicians (AI, SLG) overseeing nurse or physician abstraction.

Demographic information was collected by an electronic facesheet, with gender, race, or ethnicity self-reported. Academic centers were defined as quaternary referral centers with accredited residency, fellowship, and medical student programs. Nursing home or rehabilitation patients, if diagnosed prior to hospitalization, were defined as ambulatory patients. Comorbidities were defined as diagnosed prior to hospitalization for COVID-19. History of hypertension, diabetes, chronic lung disease (COPD or asthma), cancer, coronary artery disease, cerebrovascular disease, renal failure, and rheumatologic disease were abstracted from provider notes or medical history sections found within the EHR. If not listed in the patient’s record, the comorbidity was recorded as absent.

Presenting clinical data was abstracted from thorough review of unstructured notes as well as structured data. Hospital readmissions were included in the original admission, with baseline data used from the initial hospitalization. If multiple positive or indeterminate results were found in a patient’s record for SARS-CoV-2, the first positive test defined the date of diagnosis.

### Potential predictive variables

Twenty-two factors were considered as potentially predictive of mortality (Table 1). Of these 21 are categorical and 19 of these are dichotomous. Age is the only continuous factor. Multiple prior analyses have suggested that age is a strong predictor of COVID-19 related mortality [1–3]. The function of age that best represents its impact on mortality however is not clear. Although a step function categorized within intervals of age is a potential variable, risk can increase greatly even over as little as 5 years. Therefore, before incorporating age into the statistical procedure described below we tabulated mortality rate into 20 5-year intervals ranging from 0–4 to 100–104 and chose the power function of age that fit the data best in the sense of least squares, choosing among powers 1.0 (linear), 1.2, 1.4, etc.

**Table 1:** Results of stepwise proportional hazards modeling in training set to determine factors for inclusion in risk score.

Factors considered	Percent missing, training set	Step 1 elimi-nated	Step 2 elimi-nation order	Included in Risk Score	LogWorth = –log(p-value)	Coefficient in Risk Score
Age^3	0.0			√	27.10	4.8939e–6
Gender	0.0	√				
Current or former smoker	13.2		7			
Respiratory rate ≥ 25/min*	2.7			√	7.66	1.3711
Oxygenation <94% sat	1.4			√	8.69	1.1623
Asthma	2.5	√				
COPD	2.5		4			
Diabetes	1.4		8			
Insulin use	4.4		1			
Bradycardia on admission**	1.0		5			
Hypertension	1.0			√	1.38	0.4736
Hypotension on admission***	0.8		10			
Coronary disease	2.3			√	2.24	0.6362
Stroke	2.3		6			
Heart failure	2.6		2			
Arrhythmia	2.3		11			
Cancer	2.2		3			
Renal failure	2.0			√	2.24	0.8520
Advanced liver disease	2.8	√				
Rheumatologic disorder	2.8	√				
HIV or hepatitis	3.6		9			
Inflammatory bowel disease	2.4	√				

* 25 breaths/minute chosen as clearest delineator of risk.

** Initial heart rate < 60/min.

*** Initial SBP < 90 mmHg or DBP < 60 mmHg.

### Outcome measure

The primary outcome measurement was death due to any cause within 40 days of hospital admission. Patients alive at day 40 in the hospital as of May 29, 2020 or discharged alive from the hospital were censored on day 40.

### Variable selection and score construction

We randomly selected two equally sized halves of the dataset. We built the RS on the first half (the training set) and validated it on the second half (the confirmation set). Restricting to the training set we evaluated whether each of the 22 factors listed in Table 1 contributed to mortality risk in the context of the other factors. To reduce 22-dimensional risk information into a univariate risk score we proceeded in two steps. Step 1 considered each of the factors in a univariate proportional hazards model. Factors that were not statistically significant (p-value > 0.05) were dropped from further consideration for the RS. Step 2 considered the remaining factors in a multivariate proportional hazards model. Factors that were not statistically significant in this model were dropped one at a time, with the least significant factor (largest p-value) dropped from further consideration. The analysis was redone with that factor eliminated from the model. This iteration stopped when all the factors in the model were statistically significant. The RS is the final multivariate model standardized so that its range is from 0 to 10 for all possible patient risk profiles (with maximum age 104). The final step is to calculate the risk ratio (and its 95%) confidence interval) per unit of the RS in a univariate proportional hazards regression.

### Risk model validation

The model was subsequently validated on the second half of the patient cohort (confirmation set). The primary conclusion of the model building exercise is the statistical significance level of the RS built from the Training Set when applied to the Confirmation Set. As an additional and more detailed confirmation we compared actual mortality within each of 20 equal-sized bins of RS values.

### Patient-level mortality risk model

We converted the RS into a patient-level predictive probability of 40-day mortality based on the combined dataset. A product of a proportional hazards regression is “baseline mortality at the mean.” Its value at day 40, defined as “BMM40”, is the probability of death by day 40 for an individual patient whose RS equals the mean. BMM40 is calculated for patients with arbitrary RS under the proportional hazards assumption. Namely, the probability of mortality by day 40 is $PM40={1-(1-BMM40)}^{{RR}^{RS-MeanRS}}$ where RR is the risk ratio for one unit of increase in RS.

However, proportional hazards is a strong assumption. If the assumption does not provide a reasonable fit to the actual mortality rates over the full range of RS then for the patient-level mortality risk model we will instead use an empirically derived estimate of PM40 by fitting a polynomial to the actual mortality within the 20 equally sized bins of RS described above, using the combined datasets (training and confirmatory).

Statistical calculations were carried out using JMP^®^, Version 15 (390308). SAS Institute Inc., Cary, NC, 1989–2019.

## Results

### Baseline characteristics and outcomes of hospitalized COVID-19 patients

Data on 3478 hospitalized patients were abstracted for this study. 3308 subjects met inclusion criteria, excluding 43 patients who were pregnant, 88 on clinical trials, and 39 with a death within 1 day of admission. 185 had insufficient data available regarding discharge status for analysis, leaving a final cohort of 3123. Table 2 shows the marginal distributions of outcomes for baseline characteristics and potential risk factors. The median age of the entire cohort was 63 years (interquartile range 51 to 74) with a male predominance (60%). African-Americans comprised 11% of the study. Some comorbidities were common with 53% having hypertension and 31% diabetes. Most comorbidities were rare but 42% of all patients had 3 or more chronic conditions or risk factors. 127 patients (4%) were admitted to the ICU within the first day of hospitalization. Oxygen saturation below 94% was identified in 41%. When measured and recorded in the electronic health record, inflammatory markers were elevated with serum ferritin >1500 ng/mL in 26% and d-dimer >1 mcg/mL in 78% of patients. Therapies varied at the multiple hospitals but included hydroxychloroquine in the majority of patients, tocilizumab in over 200 critically ill patients, high dose corticosteroids in most ICU patients, remdisivir in selected patients on trial, and prone ventilation.

**Table 2:** Marginal baseline characteristics and outcomes (%).

Factors	All	Deaths	Alive
Overall	3123	713 (23)	2410 (77)
Race distribution			
African-American	341 (11)	65 (19)	276 (81)
Asian	136 (4)	35 (26)	101 (74)
Caucasian	1584 (51)	424 (27)	1160 (73)
Hispanic	613 (20)	114 (19)	499 (81)
Other	332 (11)	52 (16)	280 (84)
Missing	117 (4)	23 (20)	94 (80)
Age Median (IQR)	63 (51 to 74)	76 (66 to 85)	60 (48 to 71)
Age distribution			
0 to 9	11 (<1)	0 (0)	11 (100)
10 to 19	13 (<1)	0 (0)	13 (100)
20 to 29	85 (3)	4 (5)	81 (95)
30 to 39	223 (7)	6 (3)	217 (97)
40 to 49	377 (12)	15 (4)	362 (96)
50 to 59	578 (19)	61 (11)	517 (89)
60 to 69	685 (22)	148 (22)	537 (78)
70 to 79	582 (19)	198 (34)	384 (66)
80 to 89	408 (13)	185 (45)	223 (55)
90+	161 (5)	96 (60)	65 (40)
Gender			
Female	1237	262 (21)	975 (79)
Male	1885	451 (24)	1434 (76)
Current or former smoker			
Current	113	19 (17)	94 (83)
Former	539	161 (30)	378 (70)
Non-smoker	2068	414 (20)	1654 (80)
Comorbidities and Other Potential Risk Factors			
Respiratory rate ≥ 25/min			
Yes	373 (13)	169 (45)	204 (55)
No	2677	524 (20)	2153 (80)
Oxygenation <94%			
Yes	1270 (41)	394 (31)	876 (69)
No	1803	304 (17)	1499 (83)
Asthma			
Yes	276 (9)	58 (21)	218 (79)
No	2772	637 (23)	2135 (77)
COPD			
Yes	221 (7)	91 (41)	130 (59)
No	2825	606 (21)	2219 (79)
Diabetes			
Yes	943 (31)	286 (30)	657 (70)
No	2129	418 (20)	1711 (80)
Insulin use			
Yes	581 (19)	186 (32)	395 (68)
No	2414	497 (21)	1917 (79)
Bradycardia on admission			
Yes	62 (12)	42 (68)	20 (32)
No	3023	678 (22)	2345 (78)
Hypertension			
Yes	1649 (53)	527 (32)	1122 (68)
No	1441	181 (13)	1260 (87(
Hypotension on admission			
Yes	491 (16)	172 (35)	319 (65)
No	2603	531 (22)	2072 (78)
Coronary disease			
Yes	448 (15)	177 (40)	271 (60)
No	2607	518 (20)	2089 (80)
Stroke			
Yes	145 (5)	63 (43)	82 (57)
No	2906	632 (22)	2274 (78)
Heart failure			
Yes	227 (7)	107 (47)	120 (53)
No	2816	588 (21)	2228 (79)
Arrhythmia			
Yes	269 (9)	125 (54)	144 (54)
No	2779	572 (21)	2207 (79)
Cancer			
Yes	357 (12)	122 (34)	235 (66)
No	2699	575 (21)	2124 (79)
Renal failure			
Yes	216 (7)	101 (47)	115 (53)
No	2835	597 (21)	2238 (79)
Advanced liver disease			
Yes	26 (1)	12 (46)	14 (54)
No	3013	678 (23)	2335 (77)
Rheumatologic disorder			
Yes	89 (3)	31 (35)	58 (65)
No	2952	663 (22)	2289 (78)
HIV or hepatitis			
Yes	151 (5)	38 (25)	113 (75)
No	2972	675 (23)	2297 (77)
Inflammatory bowel disease			
Yes	25 (1)	7 (28)	18 (72)
No	3022	687 (23)	2335 (77)
Number of above comorbidities and other potential risk factors			
0	419 (15)	21 (5)	398 (95)
1	654 (23)	67 (10)	587 (90)
2	577 (20)	101 (18)	476 (82)
3	423 (15)	119 (28)	304 (72)
4	319 (11)	117 (37)	202 (63)
5	218 (8)	93 (43)	125 (57)
6+	218 (8)	118 (54)	100 (46)

In the training cohort (n = 1561), as of May 29, 2020 there were 336 (22%) deaths, 1066 (68%) successful hospital discharges, and 159 (10%) remained at risk while hospitalized and were censored. In the confirmation cohort (n = 1562), there were 377 (24%) deaths, 1053 (67%) successful hospital discharges, and 132 (8%) remained at risk while hospitalized.

### Importance of age-cubed as a predictor of COVID-19 mortality

**Fig 1:**
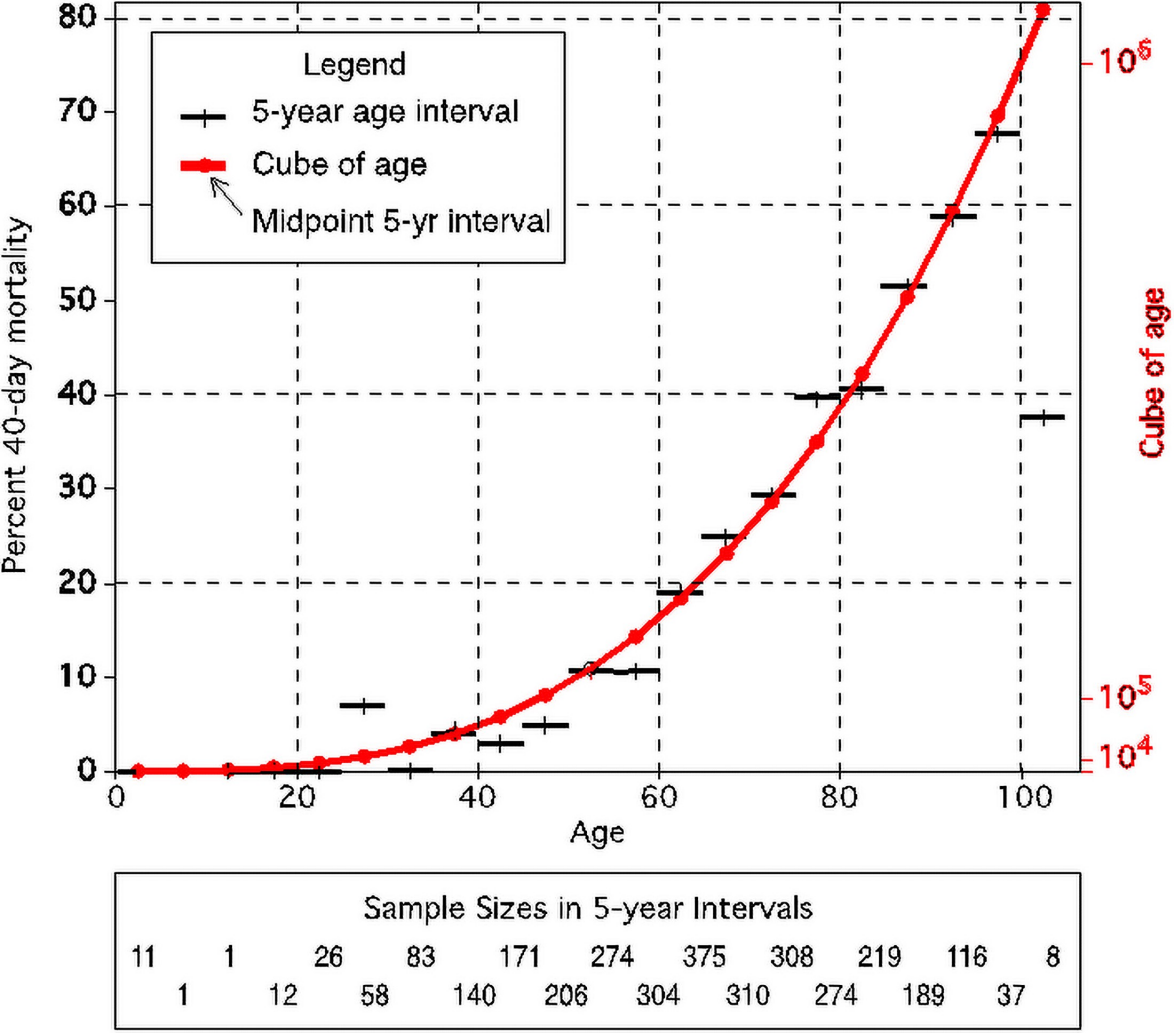
Relationship between age cubed and 40-day mortality.

Advanced age was the strongest predictor of 40-day mortality. However, the relationship was nonlinear. After tabulating mortality rates into 20 separate 5-year intervals, the best fit using a least squares model was the cube of age (i.e., age^3^) and this functional form was used in the risk model development below (Fig 1).

### Predictor selection

As shown in Table 1, 22 factors were considered in developing the mortality risk score model, with 17 factors passing step one by demonstrating in a univariate proportional hazard model a significance level less than 0.05. These factors were entered into the multivariate proportional hazards model, and after serially eliminating the least significant factors in the order shown in Table 1, 6 factors remained in the mortality risk model: age^3^, respiratory rate ≥25/minute upon hospital presentation, oxygenation <94% on hospital presentation, and pre-hospital comorbidities of hypertension, coronary artery disease, or chronic renal disease.

### COVID-19 mortality risk score

The risk score for mortality was constructed utilizing the coefficients from the final multivariate proportional hazards model:

$$\text{RS}=4.8939\text{e}-6*{\text{age}}^{3}\text{+}1.3711*\text{resp}+1.1623*\text{oxy}+0.4736*\text{hyp}+0.6362*\text{cad}+0.8520*\text{renal};$$

where age is the age in years as stated on the day of hospitalization, resp equals 1 if the respiratory rate is ≥25/minute on admission (and 0 if less), oxy equals 1 if the oxygenation level is <94% on room air upon admission (and 0 if greater), hyp equals 1 if the patient has a pre-existing hypertension comorbidity (and 0 if not), cad equals 1 if the patient has a pre-existing coronary artery disease comorbidity (and 0 if not), and renal equals 1 if the patient has a pre-existing chronic renal insufficiency comorbidity (and 0 if not). The coefficients were standardized so that the smallest possible Risk Score is 0 and the largest possible is 10. As shown in Table 3, a one unit increase in the risk score resulted in an increased risk ratio for mortality by day 40 after hospitalization of 1.80 (95% CI, 1.72, 1.87) in the combined dataset. Few patients had scores above 7, leading to an unstable model above this cutoff.

**Table 3:** Cox risk ratios for 1 unit increase in risk score.

Dataset	Sample size	Number of deaths	Risk Ratio (95% C.I.)	p-value
Training	1561	336	1.82 (1.72, 1.94)	< 0.0001
Confirmation	1562	377	1.77 (1.67, 1.88)	< 0.0001
Combined	3123	713	1.80 (1.72, 1.87)	< 0.0001

### Validation of risk score

**Fig 2:**
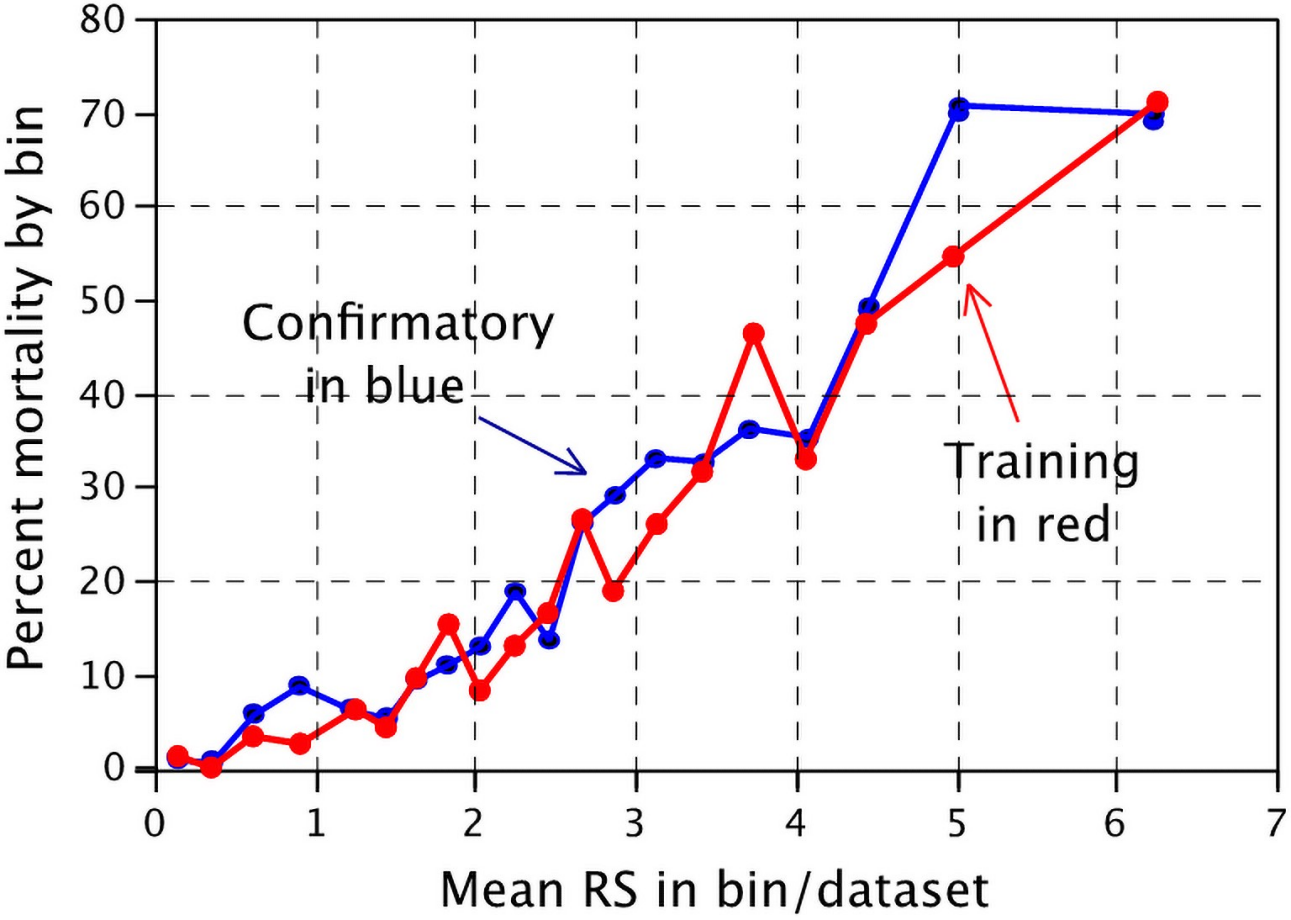
40-day mortality based on risk score. 40-day mortality among COVID-19 patients in the training and confirmatory sets as divided into bins of 20 patients of ascending mean Risk Scores. Bins 6, 11, and 16 contain 157 patients each and all other bins have 156 patients.

As shown in Table 3 the risk ratio for 40-day mortality in the training and confirmatory cohorts are similar, but with a slight and nonsignificant diminution of effect in the confirmation cohort, as expected. Each cohort was subsequently divided into 20 bins (156 patients each, except 3 bins of 157 patients) based on ascending means of risk scores. As shown in Fig 2, the percent mortality by bin increased in similar fashions in the training and confirmatory cohorts.

### Construction of model prognostic of an individual risk of dying within 40 days of COVID-19 hospitalization

**Fig 3:**
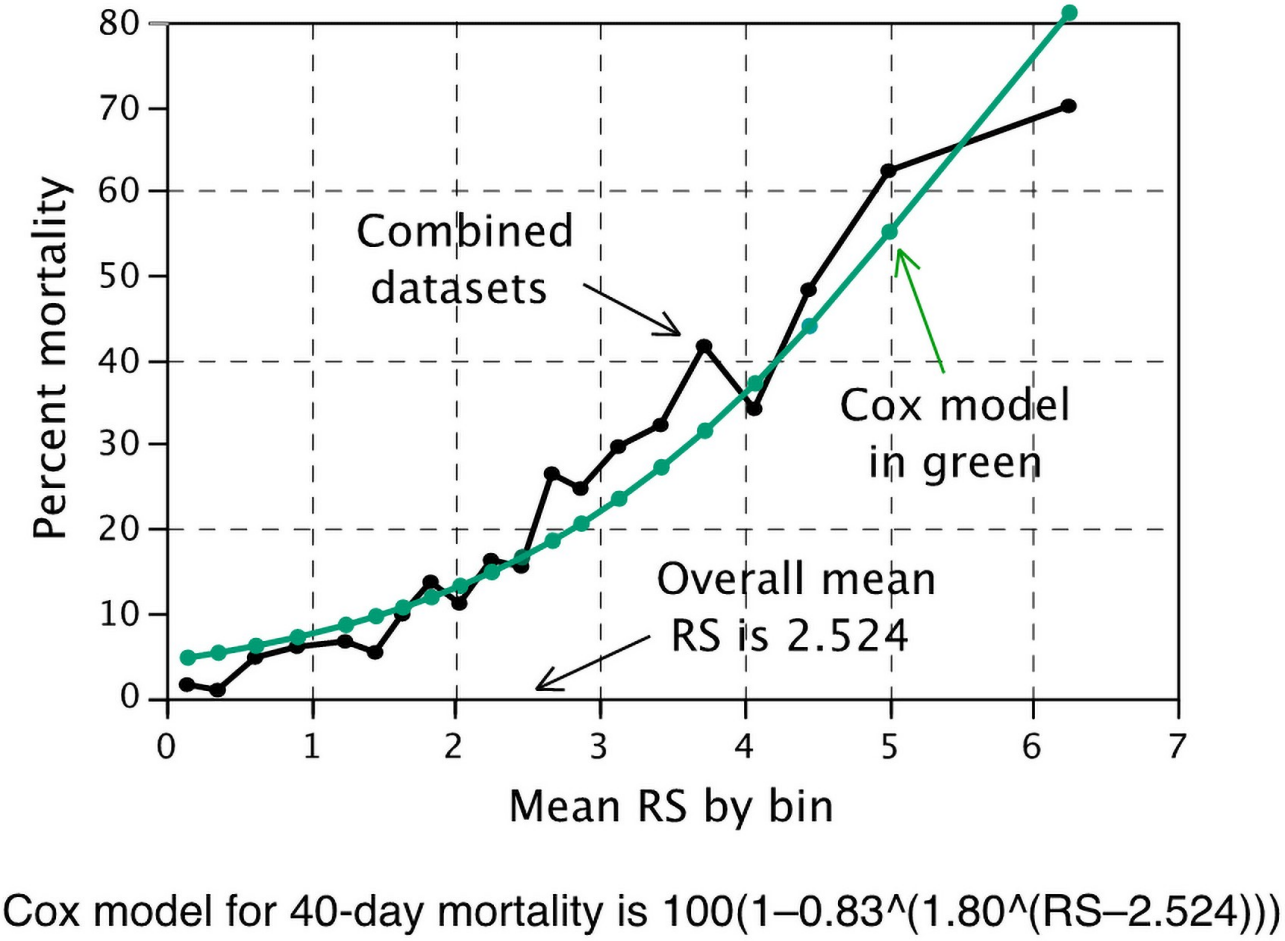
Patient mortality risk by day 40 using a proportional hazards model.

To translate the risk score to a patient-specific probability of mortality within 40 days of hospitalization for COVID-19 we utilized a proportional hazards model as described above. However, this method tended to overestimate risk for low-risk patients and underestimate risk for higher risk patients (Fig 3).

**Fig 4:**
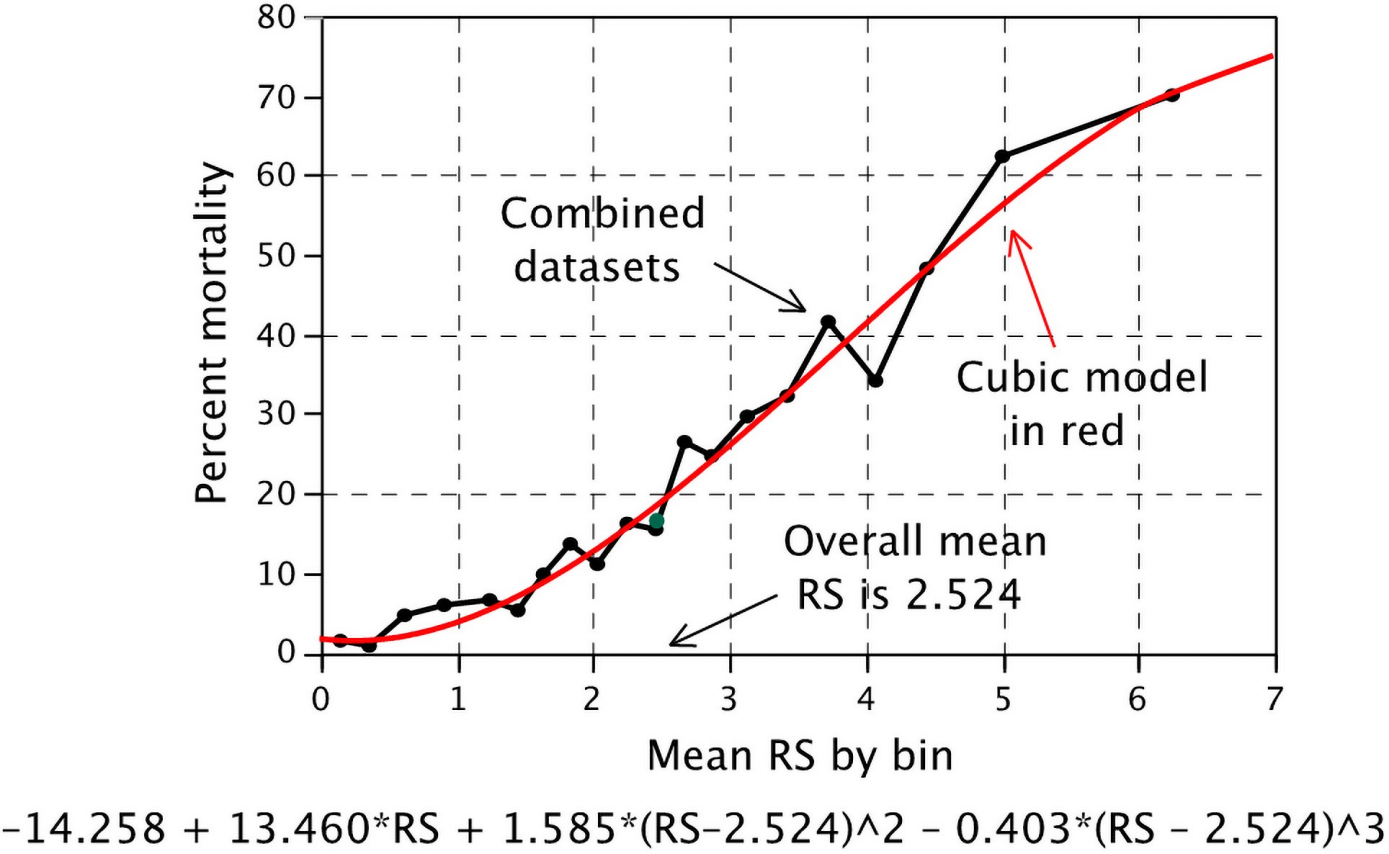
Patient-specific risk of mortality by day 40 using actual mortality in datasets.

Therefore, we used an empirically derived estimate of day-40 mortality by fitting a polynomial to actual mortality within the 20 equally sized bins of RS described above, using the combined datasets (training and confirmatory). This yielded a better fit for the model, as shown in Fig 4. Thus the patient-specific day-40 risk of mortality was defined as:

$$-14.258+13.460*\text{RS}+1.585*{\left(\text{RS}-2.524\right)}^{2}-0.403*{\left(\text{RS}-2.524\right)}^{3}.$$

### On-line calculator

An online calculator based on risk score has been developed to allow clinicians to enter the values of the 6 variables required for the risk score with automatic calculation of the projected 40-day COVID-19 mortality. (Available at www.HackensackMeridianHealth.org/CovidRS).

## Discussion

Using an observational database composed of 3123 hospitalized patients throughout the state of New Jersey we have developed and internally validated a COVID-19 mortality risk model. The risk score reduces patient age, presenting respiratory rate and oxygen saturation, and comorbidities (pre-existing hypertension, coronary artery disease, and chronic renal disease) into a single covariate with a hazard ratio of 1.80 for one unit of increase in risk score. This risk score can then be utilized to prognostic an individual’s risk of dying within 40 days of hospitalization for COVID-19 based on observed patient outcomes in our cohort.

Multiple case series have drawn associations between individual factors and poor survival outcomes [12]. In a large cohort of COVID-19 patients in Italy, Di Castelnuovo et al. found that impaired renal function, elevated C-reactive protein, and advanced age were major predictors of in-hospital death [13]. Advanced age has consistently emerged as the strongest predictor of outcome, a finding that was also observed in our model [1, 2, 12]. Interestingly, our prognostic features, apart from age, differ from the Chinese risk score model developed by Liang et al. [9]. A contributing reason is that confirmation of the prognostic value of any given comorbidity variable (such as hypertension, obesity, and coronary artery disease) is problematic as most comorbidities are more common in elderly populations. That is, these comorbidities are highly correlated with age and also with each other. To minimize the effects of such multicollinearity, we used a stepwise model to build a single risk score, one that includes components to the extent that they contribute independently to risk.

Our prognostic mortality model, which yields a quantitative risk of 40-day survival, could be invaluable for individual patient assessment and treatment planning, evaluation of new therapeutics, and facilitation of public health resource allocation. We followed TRIPOD guidelines in the reporting of our multivariable model [14]. Our sample size (>3000 patients) included significant patient populations that reached known outcomes (including death in over 700 patients) with less than 10% still at risk in the hospital within 40 days. Nonetheless, our study suffers from common limitations of observational reviews including missing data in the electronic health records, and lack of complete documentation. In addition, hospitalized patients are not representative of the population infected with SARS-CoV-2. Moreover, our study population may not well represent patient populations infected with variants of SARS-CoV-2 that have developed and spread since the period of our study.

Additionally, our risk-score model, although validated on an internal second cohort, requires external validation in a cohort of patients treated outside New Jersey to confirm generalizability. Furthermore, our model was not adjusted for treatments. During the study timeframe the majority of the SARS-CoV-2 directed care was supportive, hydroxychloroquine, and/or tocilizumab [15, 16]. Remdesivir anti-viral therapy, corticosteroids, and prone ventilatory positioning was common. As more effective therapies are developed the mortality model will need to be revised. Indeed, our risk score may serve as baseline risk to be updated based on a treatment’s efficacy. It may also serve as an indicator of patient subpopulations that may benefit from a particular therapy or as a covariate in judging the effectiveness of new therapies. Using a single risk score avoids the pitfalls of multicollinearity in assessing observational data, but improves upon propensity score modeling by applying risks to each individual subject rather than the entire population.

In summary, comorbid conditions are common among patients hospitalized for COVID-19. However, 6 features at the time of hospital presentation can be utilized to generate a single covariate helpful in prognosticating an individual’s risk of dying within 40 days of a COVID-19 related hospitalization. Our model also confirms that age is the single most important characteristic for survival from this infection. Our risk model is available online (www.HackensackMeridianHealth.org/CovidRS) and may assist in patient assessments, evaluation of new therapeutics, and public healthcare projections.
